# Neonatal cellular and gene therapies for mucopolysaccharidoses: the earlier the better?

**DOI:** 10.1007/s10545-015-9900-2

**Published:** 2015-11-17

**Authors:** Shunji Tomatsu, Isabella Azario, Kazuki Sawamoto, Alice Silvia Pievani, Andrea Biondi, Marta Serafini

**Affiliations:** Department of Biomedical Research, Alfred I. duPont Institute Hospital for Children, Wilmington, DE USA; Dulbecco Telethon Institute at Centro Ricerca M. Tettamanti, Department of Paediatrics, University of Milano-Bicocca, San Gerardo Hospital, via Pergolesi, 33, 20900 Monza, MB Italy; Centro Ricerca M. Tettamanti, Department of Paediatrics, University of Milano-Bicocca, Via Pergolesi, 33, Monza, 20900 Italy; Skeletal Dysplasia Lab, Department of Biomedical Research, Nemours/Alfred I. duPont Hospital for Children, 1600 Rockland Rd., Wilmington, DE 19899-0269 USA

## Abstract

Mucopolysaccharidoses (MPSs) are a group of lysosomal storage disorders (LSDs). The increasing interest in newborn screening procedures for LSDs underlines the need for alternative cellular and gene therapy approaches to be developed during the perinatal period, supporting the treatment of MPS patients before the onset of clinical signs and symptoms. The rationale for considering these early therapies results from the clinical experience in the treatment of MPSs and other genetic disorders. The normal or gene-corrected hematopoiesis transplanted in patients can produce the missing protein at levels sufficient to improve and/or halt the disease-related abnormalities. However, these current therapies are only partially successful, probably due to the limited efficacy of the protein provided through the hematopoiesis. An alternative explanation is that the time at which the cellular or gene therapy procedures are performed could be too late to prevent pre-existing or progressive organ damage. Considering these aspects, in the last several years, novel cellular and gene therapy approaches have been tested in different animal models at birth, a highly early stage, showing that precocious treatment is critical to prevent long-term pathological consequences. This review provides insights into the state-of-art accomplishments made with neonatal cellular and gene-based therapies and the major barriers that need to be overcome before they can be implemented in the medical community.

## Introduction

### Background

Mucopolysaccharidoses (MPSs) comprise a group of lysosomal storage disorders (LSDs) having in common the inherited deficiency of a particular lysosomal enzyme and the subsequent accumulation of undigested glycosaminoglycans (GAGs). GAG storage results in loss of cellular functions, tissue damage, and organ dysfunction accounting for clinical signs and symptoms observed in patients. Clinical manifestations include mental retardation, skeletal dysplasia, corneal clouding, abnormal facies, coarse hair, hernia, hepatosplenomegaly, respiratory and valvular heart diseases, and abnormal joint mobility (Neufeld and Muenzer 2001). At the skeletal level, MPS patients develop a characteristic dysostosis multiplex due to the progressive storage of GAGs in the bones, especially chondroitin sulfate, dermatan sulfate, and/or keratan sulfate (Neufeld and Muenzer 2001).

### Difficulty in early diagnosis

It is extremely difficult to diagnose MPS patients at birth, and even at the onset of clinical disease. For instance, most MPS I patients may not have MPS-specific signs and symptoms at birth although umbilical and/or inguinal hernia is common. Diagnosis of Hurler syndrome, the most severe form of MPS I, is commonly made between 4 and 18 months of age. A combination of symptoms, as skeletal deformities, recurrent respiratory infections, inguinal and umbilical hernias, coarse facial features, hepatosplenomegaly, and enlarged tongue leads to initial medical attention. Without appropriate treatment, the life expectancy of patients with Hurler syndrome is limited: the median survival is less than 10 years, with only rare survivors beyond 10 years. Diagnosis of other types of MPS may be delayed more than MPS I since clinical manifestations occur later.

### Early treatments

Currently, several treatments such as enzyme replacement therapy (ERT), hematopoietic stem cell transplantation (HSCT), and gene therapy are being evaluated for MPSs. ERT and HSCT are clinically available, and gene therapy is under clinical trials for some types of MPSs. ERT, based on the administration of recombinant enzyme, is actually being employed in patients with MPS I (Kakkis et al 2001), MPS II (Muenzer et al 2002, 2006), MPS IVA (Hendriksz et al 2014), MPS VI (Harmatz et al 2008), and MPS VII (Fox et al 2015). Indeed, the treatment with ERT has shown demonstrable benefits, especially in joint mobility, respiratory functions, decrement in organs volumes, and reduction in urinary GAG excretion (Muenzer 2014). However, current ERT for MPSs has severe limitations, such as an inadequate effect on skeletal and neurological symptoms (Connock et al 2006; Muenzer 2014; Rohrbach and Clarke 2007), a rapid clearance from the circulation, and immune reactions, due to the development of anti-enzyme antibodies (Dickson et al 2008; Kakkis et al 2001). Several demonstrations have shown that the earlier ERT is performed in animal models and human patients, the better the outcome is (Dierenfeld et al 2010; Sands et al 1994; Tomatsu et al 2015). Similar evidence comes from the follow-up analysis of MPS I patients treated with HSCT. Indeed, a retrospective analysis could demonstrate superior long-term clinical outcome for patients with MPS I when HSCT was performed early in life (Aldenhoven et al 2015; Boelens et al 2013). HSCT of MPS I patients (transplanted with a median age of 16 months) improves their quality of life and neurocognitive development, albeit the therapeutic effect on bone lesions remains limited (Aldenhoven et al 2015; Boelens et al 2013). The musculoskeletal manifestations are still deteriorated and provide an impact on the quality of life in most transplanted patients with MPSs (Aldenhoven et al 2008). One explanation could be the limited penetration of the expressed enzyme into musculoskeletal tissues (Field et al 1994). Another possibility is that irreversible bone damage has already occurred prior to the time of the transplant. Considering the current experimental and clinical data on both the available treatment options, it is likely that an early intervention in patients with MPSs could be critical for obtaining a higher degree of correction. It is notable that MPSs may represent the ideal model for elucidating these aspects since 1) they are monogenic diseases, 2) several animal models are available, and 3) the successful restoration of even a low level of enzyme activity is expected to be sufficient to correct or improve the disease. For these reasons, MPSs are conceived as conditions suitable for the evaluation of innovative early therapeutic strategies.

In particular, this review focuses on the scientific evidence demonstrating that cellular and gene therapies in the neonatal period provide a real therapeutic perspective for MPS disorders.

## Neonatal hematopoietic stem cell transplantation

Cellular therapy is relevant for some forms of MPSs. HSCT has been shown to be one of the most effective treatment strategies for patients with Hurler syndrome. In particular, the use of various HLA-matched hematopoietic stem cell sources (peripheral blood, bone marrow of unrelated donors or cord blood) has contributed to offer a transplantation strategy to a significant number of patients (Aldenhoven et al 2008; Peters et al 2003; Rovelli 2008; Yasuda et al 2015).

In general, early HSCT improves the pathology in all organs although bone lesions have a lesser impact compared with visceral organs. Thus, HSCT alleviates most clinical manifestations in these patients, probably due to the migration of the transplant-derived cells into organs, where they can secrete the functional enzyme and clear the lysosomal storage leading to the correction of the metabolic defect. However, the effect of HSCT on the orthopedic manifestations is limited likely by the poor penetration of the donor cells into the musculoskeletal tissues (Aldenhoven et al 2008; Field et al 1994). It is likely that HSCT provides more circulating enzyme to bone rather than directly affecting bone by migration of the cells.

Furthermore, the recovery of the patients’ skeletal phenotype produced by HSCT could be incomplete likely because bone abnormalities are irreversible at the time of the transplant. For this reason, the impact of HSCT could still benefit from further improvements, as the use of different stem cell sources and/or alternative transplant procedures. In particular, a neonatal cellular therapy approach may hold more promise, considering that it would allow preventing the progressive disease manifestations, which develop during early stage.

Several reports have evaluated if the perinatal infusion of stem cells of hematopoietic origin could ameliorate the most prominent clinical features in MPS animal models (Table 1). Soper et al published a study describing a non-ablative neonatal marrow transplantation model in MPS VII mice (Soper et al 2001). Despite low-level engraftment of donor cells, MPSVII mice treated with bone marrow transplantation (BMT) in neonatal life have shown several improvements, including extension of life span, reduction of lysosomal storage in multiple tissues, and amelioration of bone parameters (Sands et al 1993; Soper et al 2001). Successive neonatal BMT procedure in the MPS VII mice also proved that the aberrant electrocardiogram and many of the progressive heart lesions were corrected in the long-term (Schuldt et al 2004). Furthermore, BMT in newborn MPS VII mice has led to the prevention of early hearing loss and to an improvement in the histopathology of the ear (Sands et al 1995). In the central nervous system (CNS), a consistent reduction in the amount of storage in the meninges and glial cells, but not in the neocortex, hippocampus, and cerebellum has been reported (Soper et al 2001). It is noteworthy that the CNS function of MPS VII mice, transplanted after a myeloablative irradiation and evaluated with two behavioral tests, was not ameliorated in transplanted mice (Bastedo et al 1994). Similarly, in a mouse model of MPS IIIA, neonatal BMT did not affect neuropathological storage (Lau et al 2013). Likewise, BMT of MPS IIIB mice performed at 2–4 days of age after irradiation did not show any evident improvement in the brain (Heldermon et al 2010). The authors concluded that BMT does not correct the CNS abnormalities of transplanted mice, even though the lack of improvement could also be attributed to the transplant procedure, in particular to radiation-induced toxicity in CNS or to the low engraftment of donor cells.

**Table 1:** Neonatal cell therapy and gene therapy in MPS animal models: overview from the literature

Therapy		Disease	Model	Administration	Source	Conditioning			
						None	TBI	Drugs	Other
Cell therapy		MPS I	mouse	IV	BM			Pievani et al 2015	
				CNS	hBM-MSC	Nan et al 2012			
		MPS III A	mouse	IV	BM		Lau et al 2013		
		MPS III B	mouse	IV	BM		Heldermon et al 2010		
		MPS VI	rat	IP	BM		Simonaro et al 1997		
		MPS VII	mouse	IV	BM	Schuldt et al 2004; Soper et al 2001	Bastedo et al 1994; Sands et al 1993, 1995		Lessard et al 2006*
Therapy		Disease	Model	Administration	Source	Vector			
						Adeno	AA	Retro	Lenti
Gene therapy	in vivo	MPS I	mouse	IV			Hartung et al 2004	Baldo et al 2013; Chung et al 2007; Liu et al 2005; Ma et al 2008; Ponder et al 2006	Kobayashi et al 2005
				CNS			Wolf et al 2011		
			cat	IV				Ponder et al 2006	
			dog	IV				Herati et al 2008; Traas et al 2007	
				CNS			Hinderer et al 2015		
		MPS III B	mouse	IV					Heldermon et al 2013
				CNS			Heldermon et al 2010, 2013		
		MPS VI	rat	IV			Tessitore et al 2008		
				IM			Tessitore et al 2008		
			cat	IV			Tessitore et al 2008	Ponder et al 2012	
				IM			Tessitore et al 2008		
		MPS VII	mouse	IV		Kamata et al 2003; Kanaji et al 2003	Daly et al 1999b, 2001; Elliger et al 1999	Mango et al 2004; Xing et al 2015; Xu et al 2002b	Derrick-Roberts et al 2014
				CNS			Elliger et al 1999; Passini et al 2003		
				IM			Daly et al 1999a		
			dog	IV				Bigg et al 2013; Herati et al 2008; Mango et al 2004; Metcalf et al 2010; Ponder et al 2002; Sleeper et al 2004; Smith et al 2012; Wang et al 2006; Xing et al 2013; Xu et al 2002a	
	ex vivo	MPS VI	cat	IV	BM/NBB			Simonaro et al 1999	
		MPS VII	mouse	CNS	hNSC			Meng et al 2003	
				IP	hBM-MSC				Meyerrose et al 2008

*AA* adeno-associated virus, *TBI* total body irradiation, *IV* intravenous, *CNS* central nervous system, *IM* intramuscular, *IP* intraperitoneal, *BM* bone marrow, *NBB*, newborn blood; hNSC, human neural stem cells; hBM-MSC, human bone marrow mesenchymal stem cells

*costimulatory blockade anti-CD40L mAb and CTLA-4Ig

With the aim of evaluating the effect at the skeletal level, neonatal BMT has recently been tested in a MPS I mouse model (Pievani et al 2015). This study adopted a busulfan-based conditioning followed by a syngeneic BMT in the first days of life. Busulfan is a standard chemotherapy agent used in patients in combination with other drugs as a conditioning prior to HSCT, especially in leukemia, lymphoma, myeloproliferative disorders, and MPSs. The use of busulfan in a neonatal experimental setting is an element of interest since it allows a better engraftment of donor cells in the brain compared with irradiation, which could also provide an effect on the neurological manifestations of the disease (Wilkinson et al 2013). Furthermore, by using this experimental regimen with busulfan in MPS I mice (Pievani et al 2015), the extent of engraftment obtained was high, and the successive clinical improvement was very encouraging. The replacement of the hematopoiesis resulted in an increase in alpha-L-iduronidase (IDUA) activity in peripheral organs and, consequently, clearance of GAGs in plasma and various tissues. At 37 weeks of age, the reconstitution of normal hematopoiesis in MPS I mice was associated with a consistent amelioration of skeletal dysplasia. Radiographic analysis showed that the widths of the skull, of the zygomatic arches, and of the long bones in MPS I early-treated mice were almost normalized. The bone morphometric parameters calculated by micro-CT revealed a 40–80 % improvement in neonatally transplanted compared with untransplanted MPS I mice, approaching the values observed in WT. The authors also noticed a reduction of both hyperosteocytosis and lysosomal vacuolization in femur sections of neonatally transplanted MPS I mice.

Overall, the magnitude of improvements correlated with the extent of hematopoietic engraftment, interestingly suggesting that the early restoration of normal hematopoiesis provides a favorable impact on the bone development in MPS I. Therefore, BMT at a very early stage in life reduces signs and symptoms of MPSs in animal models by preventing their development.

## Neonatal gene therapy

Gene therapy also offers a potential therapeutic opportunity for MPSs. In theory, gene therapy for these disorders should act by providing the affected cells with enough enzyme. The mechanisms by which gene therapy could be effective include 1) the delivery of the gene directly to the cells mainly involved in these diseases and 2) the uptake by these cells of the missing enzyme secreted from other transduced cells acting as an enzyme source. Early gene transfer in the neonatal period overcomes some issues, which can occur in gene therapy performed in adulthood. First, in mature organisms affected by MPSs, the genetic defect has already caused irreversible pathological lesions mainly in bone and brain. Therefore, for MPSs and many other genetic diseases, gene therapy at birth will have a striking effect to arrest disease progression. In addition, if gene therapy is administered in adulthood, an immune response may rapidly eliminate transgenic protein expression precluding any favorable effect. In contrast, with an early intervention, the possibility to develop a vigorous immune system response toward the transgenic protein is less likely than in adulthood. Furthermore, in the case of an approach based on the use of genetically corrected hematopoietic stem cells, the autologous setting reduces the risks related to an allogeneic transplant (graft versus host disease, GVHD) and provides potential advantage to patients lacking an HLA-matched donor.

In addition, a study in the MPS VII mouse model has shown that, as the disease progresses, more genes present altered expression, and this may account for the complex clinical phenotype. Only some of those changes in gene expression normalize when the treatment is initiated in animals with established disease, and this observation further supports the need of an early intervention (Woloszynek et al 2004).

**Fig. 1:**
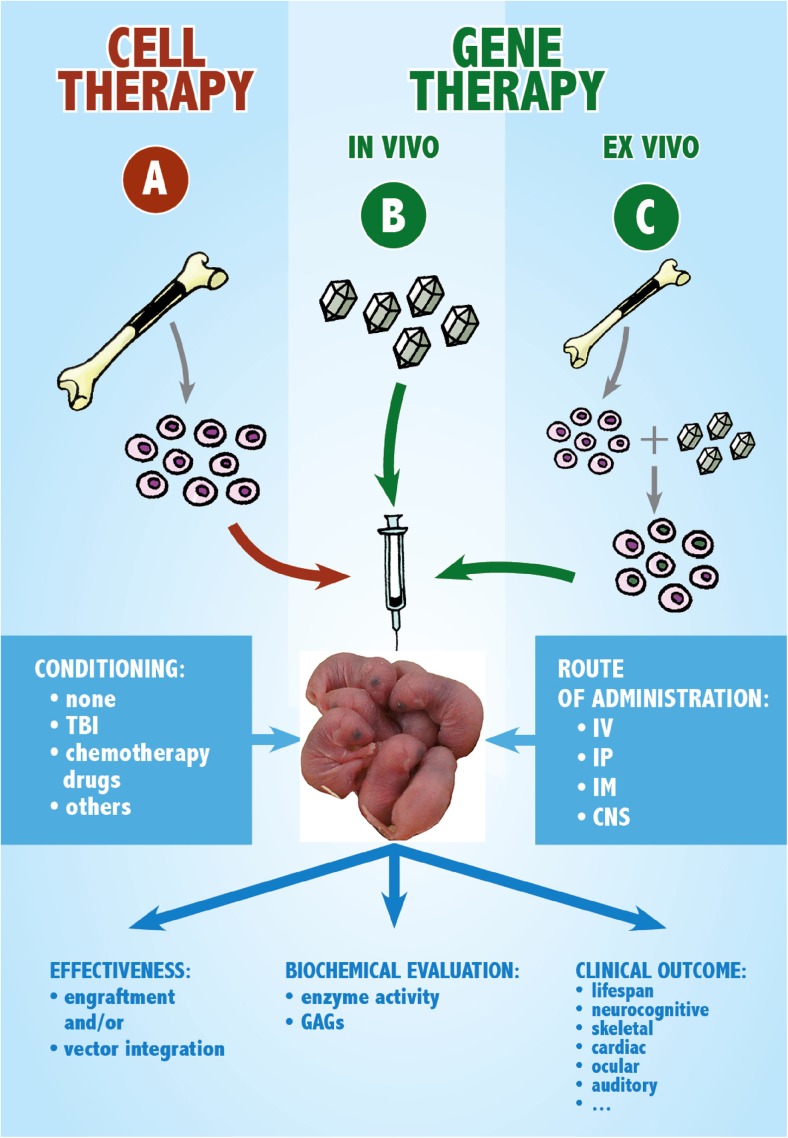
Scheme of the approaches employed for the neonatal therapy of MPS animal models. **a** Cell therapy. Cells from the bone marrow (or alternative stem cell sources) of a healthy donor are collected and then transplanted into the affected newborn. **b** Gene therapy in vivo. A viral vector carrying a functional copy of the defective gene is injected into the organism of the affected neonate. **c** Gene therapy ex vivo. Bone marrow stem cells (or stem cells from other sources) are transduced ex vivo with a viral vector carrying a copy of the defective gene and then gene-corrected cells are transplanted into the affected neonate. Two important factors in the experimental setting are the conditioning and the route of administration. The effectiveness of the approach is evaluated, and the outcome is observed focusing on biochemical and clinical parameters. Abbreviation: TBI, total body irradiation; IV, intravenous; IP, intraperitoneal; IM, intramuscular; CNS, central nervous system (route: intraventricular or intratechal); GAGs, glycosaminoglycans

The two primary categories of somatic gene therapy consist of (1) the in vivo infusion of viral vector particles with the aim of transferring normal cDNA to the affected cells enabling them to express the missing protein or (2) the ex vivo transduction of patient’s cells which could be subsequently infused (Fig. 1). The first form of gene therapy is called in vivo because the gene vector is transduced to cells inside the patient’s body. In the ex vivo procedure, cells from the patient’s blood or bone marrow are cultured in the laboratory, exposed to the viral vector that is carrying the desired gene and then returned to the patient.

### In vivo neonatal gene therapy

In the last two decades, in vivo gene therapy studies in neonatal MPS mice and large animal models have been reported by using retroviral, adenoviral, lentiviral, and adeno-associated virus (AAV)-based vectors (Table 1). Systemic gene therapy could program some cells to secrete the lacking enzyme, which could be taken up by other affected cells via the mannose 6-phosphate receptor. The first report demonstrating the several advantages of an early in vivo gene therapy approach showed that intravenous AAV-mediated gene transfer in neonatal MPS VII mice may offer an efficient system to widespread correction (Daly et al 1999b). Also in a CNS-directed gene therapy approach, the injection of recombinant AAV encoding human GUSB into both the anterior cortex than the hippocampus of newborn MPS VII mice had positive effect not only on the brain histopathology, but also improved cognitive function (Frisella et al 2001). Subsequently, Hartung et al treated MPS I mice at birth with an AAV vector carrying the human IDUA cDNA and showed that AAV-IDUA gene transfer into newborn MPS I mice led to high levels of plasma and tissue enzyme activities, which were sufficient to normalize urine GAG levels and reduce lysosomal storage in a number of the main organs of treated mice (Hartung et al 2004). The effect of IDUA restoration on craniofacial and CNS parameters demonstrated significant improvements on these critical features of MPS I. Similarly, the in vivo injection of high doses of retroviral vector expressing IDUA resulted in the complete correction of biochemical and pathological evidence of disease in internal organs, bone, and brain (Chung et al 2007; Liu et al 2005). In particular, Liu et al showed that MPS I mice that received high-dose of retroviral vectors had normal echocardiograms, bone mineral density, auditory-evoked brain-stem responses, and electroretinograms (Liu et al 2005).

With the advent of lentivirus-based vector technology, Kobayashi et al estimated the possibility to perform gene therapy for MPS I by direct in vivo injection of a lentiviral vector (Kobayashi et al 2005). They compared the efficacy between newborn and young adult MPS I mice of lentiviral vector-mediated gene therapy, demonstrating a significantly greater advantage for mice treated neonatally. In particular, they showed that the neonatal administration of the lentiviral vector by a single intravenous injection led to a sustained expression of active IDUA enzyme in multiple organs, including the brain, in which vector administration resulted in transduction of neurons in the brain. Interestingly, the authors were able to show a clear advantage in mice treated as neonates compared to those treated as young adults. Indeed, the disease manifestations were only moderately improved in this latter group, but almost normalized in mice treated earlier in life. When the vector was administered at birth, the IDUA activity resulted in decreased GAGs storage, prevention of skeletal abnormalities, a more normal gross appearance, and improved survival. The extent of transduction was dose-dependent, with the liver receiving the higher level of the vector, but other somatic organs reaching almost similar levels.

All these works provided different confirmations of the effectiveness of an early gene therapy approach on MPS mouse models, independently of the type of vector used.

In recent years accumulating evidence has provided support for the hypothesis that another critical point, which can impact the efficacy of the neonatal gene therapy approach, is dependent not only on the virus type used but also on the route of administration.

The intracranial injection alone of an adeno-associated viral (AAV) vector in the mouse model of MPS IIIB resulted in improvement in lifespan, motor function, hearing, time to activity onset, and daytime activity level, but no effects on the lysosomal storage (Heldermon et al 2010). A more recent report has described a novel approach using the combination of an intracranial injection of an AAV-based vector and an intravenous treatment with a lentiviral vector, showing better clinical, histological, and biochemical features (Heldermon et al 2013).

Although the majority of gene transfer experiments for the treatment of inherited or acquired diseases have mainly been performed in mice, large animal models clearly represent an important step in the preclinical evaluation of a gene therapy approach, as their responses are probably more predictive of the results in humans. Large animals are more similar in size to a neonate offering a more sophisticated disease modeling resembling several human features.

The efficacy of neonatal gene therapy has also been tested in large MPS animal models. The first successful application of neonatal gene therapy in large animals has been described by Ponder et al, who reported the clinical improvements seen in MPS VII dogs treated with a cGUSB-expressing retroviral vector as neonates (Ponder et al 2002). In particular, little or no corneal clouding and no mitral valve thickening have been observed. Radiographically, treated dogs had fewer skeletal abnormalities, and, therefore, they could run at all planned times of evaluation. Estimating the long-term effect of this treatment on MPS VII dogs, the authors concluded that neonatal gene therapy was able to appreciably, even if not entirely, reduce bone and joint disease (Xing et al 2013). It is notable that neonatal gene therapy in MPS VII dogs was still not effective in preventing lumbar spine disease (Smith et al 2012). Similarly, in newborn MPS VI cats treated with a feline N-acetylgalactosamine 4-sulfatase-expressing retroviral vector, the results indicated, at the bone level, improvements in some aspects such as femur length, articular cartilage erosion, mobility, but not a significant effect on cervical vertebral bone length (Ponder et al 2012). Thus, the impact of neonatal gene therapy is different in different bones. It is of great interest to understand which bone is severely affected, when each bone starts to be affected by the disease or what difference is present in penetration of gene vector and its expression level in each bone.

Pivotal information can be evinced from the studies conducted in MPS I dogs (Traas et al 2007). In this work, MPS I dogs were treated at birth with a gamma retroviral vector expressing the canine IDUA, and yielded a clinical effect derived from a stably expressed and circulating enzyme without showing an immune response (Traas et al 2007). The authors speculated that the immaturity of the newborn immune system or the tolerance to canine IDUA epitopes could have contributed to prevent the production of anti-canine IDUA antibodies.

These findings on neonatal in vivo gene therapy are promising and pave the way for upcoming clinical trials, even if future studies in patients need to assess the risks and benefits of the adopted vector.

### Ex vivo neonatal gene therapy

Ex vivo gene therapy consists of two steps: 1) infecting somatic cells in vitro by viral vectors, then 2) injecting gene-corrected cells in vivo into a newborn recipient organism. Such an approach is of particular interest in the case of MPSs: in fact, it allows the transduction of the defective gene directly into recipient cells, and the subsequent transplant of transduced cells in an autologous setting, avoiding the common immune issues of allogeneic transplantation. Moreover, the viral infection often allows reaching supraphysiological levels of protein expression in corrected cells, which could never be achieved using normal donor cells as a source of enzyme. This is another apparent advantage of ex vivo gene therapy, since it has been shown in animal models and in clinic that the extent of phenotypic correction is strongly related to the levels of enzyme activity reached in the recipient organism (organs or biological fluids) as a result of the treatment (Aldenhoven et al 2015; Visigalli et al 2010).

In MPS animal models, this gene-correction strategy has mainly been performed on long-term hematopoietic repopulating cells, usually derived from bone marrow, which are cultured and transduced, and then transplanted into recipients with a standard procedure of bone marrow transplantation (Visigalli et al 2010; Wakabayashi et al 2015; Wang et al 2009; Zheng et al 2003).

Only a few papers related to neonatal ex vivo gene therapy in MPS animals have been published (Table 1). Simonaro et al described the transplantation of retrovirally transduced bone marrow (BM) or newborn blood cells in MPS VI cats, some of which were very early in life. Cells transduced with the vector carrying the cDNA for the human arylsulfatase B, the enzyme deficient in MPS VI, were successfully engrafted and persisted for a long term in the cats, while the level of enzyme activity reached was low, not sufficient to appreciate any clinical improvement. Notably, the authors employed as cell source not only BM, but also newborn blood, in an attempt to verify the feasibility of transplanting gene-corrected cord blood (Simonaro et al 1999).

Other cell types have also been employed for gene correction. In 2003, Meng et al focused on the CNS involvement in MPSs. They transduced human neural stem cells (hNSCs) with a retroviral vector, in order to express the human enzyme ß-glucuronidase at supranormal levels; then they injected corrected cells into the cerebral ventricles of immunodeficient MPS VII newborn mice. They identified the presence of hNSCs in host brains, the presence of ß-glucuronidase activity, and a reduction of lysosomal storage. Unfortunately, these effects lasted only for a short time after transplantation because human cells rapidly underwent apoptosis (Meng et al 2003).

Meyerrose et al obtained even more encouraging results. They transduced human BM-derived mesenchymal stem cells (hBM-MSCs) by a lentiviral vector, forcing them to overexpress human ß-glucuronidase. The intraperitoneal transplantation of corrected hBM-MSCs into neonatal NOD-SCID MPS VII mice leads to the engraftment of these cells in several organs and to their release of therapeutic levels of enzyme (nearly 40 % of normal in serum), detected 2 and 4 months after the transplant. As a result, the storage of GAGs and the secondary elevated activities of other lysosomal enzymes were normalized. Notably, the authors attested even a clinical amelioration since treated mice showed an improvement in retinal function (Meyerrose et al 2008).

The described approaches, with their interesting initial results, are significant proofs of principle for the application of ex vivo gene therapy in the very early treatment of MPSs.

## Advent of newborn screening programs and future neonatal therapies

### Newborn screening on MPSs

It is inevitable to establish newborn screening systems for MPS patients to allow an early diagnosis and early therapy. There are two principal methods that are being developed and pilot studies are currently underway; one is the assay which measures the deficient enzyme in each MPS disorder, and the other one is the assay which measures primary storage substrates, GAGs. Both methods will analyze newborn dried blood spots (DBSs).

Enzyme assay method can be a useful tool in newborn screening for MPSs, measuring the activity of each deficient enzyme directly. This method is highly sensitive and specific. There are several reports of enzyme assays in MPS I (Blanchard et al 2008; Wang et al 2005) and MPS II (Wang et al 2007) patients. This group and others have developed direct multiple assays of enzyme activity in DBS samples by using tandem mass spectrometry (MS/MS) for newborn screening of lysosomal storage diseases (Gelb et al 2006; Li et al 2004). The enzyme assay can provide a diagnosis of the disease directly while the disadvantage is that the method cannot differentiate the pseudodeficiency from true positive patients. The micro-fluidics methodology is also being investigated for screening DBSs and has already been applied in a full-population pilot study in Missouri (Hopkins et al 2015).

Another method is to measure primary storage materials, GAGs. Several groups have developed highly sensitive, specific, and inexpensive assay methods to distinguish patients with MPSs from healthy controls by using liquid chromatography-tandem mass spectrometry (LC-MS/MS) systems. Tomatsu et al measured heparan sulfate and dermatan sulfate levels in DBS samples from six neonatal MPS patients (four MPS I, one MPS II, and one MPS VII) and compared each GAG level from these MPS samples with that from healthy control samples (*n* = 326) in a double blind method. Both levels were markedly elevated in all six samples of MPS patients compared with the levels of control samples (Oguma et al 2007; Tomatsu et al 2010a, b, c). This group also measured 12 newborn samples with MPSs (six MPS I, one MPS II, and five MPS III) using both LC-MS/MS and high-throughput mass spectrometry (HT-MS/MS) (Shimada et al 2014). The disaccharide levels of ΔDiHS-0S and ΔDiHS-NS from DBS samples with MPS I or MPS III were markedly elevated compared with those from control newborn samples (*n* = 22). Also in the case of MPS II samples, these levels were respectively 3 and 1.5 times higher than in controls (Shimada et al 2014). De Ruijter et al have also reported that the disaccharide levels of heparan sulfate and dermatan sulfate from newborn DBS samples obtained from MPS patients (11 MPS I, one MPS II, and six MPS III) were significantly increased in all patients samples compared with controls (de Ruijter et al 2012).

Therefore, these methods of newborn screening for MPS patients can be useful tools to make an early diagnosis for at least MPS I, II, and III. This group is starting a pilot study to measure specific GAGs from a total of 200,000 newborn DBS samples and to evaluate the new assay systems for MPSs newborn screening. This method provides a suggestion of a high-risk group for MPSs and can be useful for assessing the clinical severity of the disease and monitoring the therapeutic efficacy and/or pharmacokinetics of the drug, while the second screening with enzyme assay is required for certain diagnosis.

Both methods have some limitations, such as false-positive/negative and costs in GAG assay and enzyme assay method. These issues still need to be resolved before establishing a newborn screening system for MPSs in the clinical practice.

### Future neonatal therapies

As previously mentioned, ERT is a standard therapy for MPSs and is approved or under clinical trials in many countries for MPS I, MPS II, MPS IVA, MPS VI, and MPS VII patients. Patients treated with ERT showed clinical improvement of somatic manifestations and an enhanced quality of life. However, there are several limitations: 1) ERT is least efficacious on CNS and skeletal dysplasia, 2) the enzyme has a short half-life and high clearance from the circulation, 3) continuous ERT causes immunological problems, and 4) it is very expensive.

HSCT for MPS patients has been conducted prior to ERT; however, initial attempts of HSCT were controversial because of a high mortality rate. Several results on MPS animal models suggest that skeletal deformities and impaired growth development in MPS patients should be improved if HSCT is performed at earlier stages. HSCT has a risk for the development of mortality by GVHD, infections and additional complications. It is known that the severity of GVHD is influenced by the donor match and by pre-HSCT serotherapy. Anyway, conditioning regimens for HSCT have been markedly improved in each medical facility, and well-trained staffs contribute to the least mortality of HSCT.

Busulfan is a standard chemotherapy drug usually given as a conditioning agent prior to HSCT. However, this drug may still induce severe side effects such as toxicity in lung and liver. Treosulfan (treo) is another alkylating cytotoxic agent with a supposedly less severe toxicity profile. It is most commonly used in the treatment of ovarian cancer. It is also increasingly used in HSCT, predominantly in non-malignant diseases. In European countries, treosulfan is approved and used efficiently and safely in pediatric patients before HSCT (Bernardo et al 2012; Boztug et al 2015; Slatter et al 2011; Strocchio et al 2015; Wachowiak et al 2011). Pediatric MPS patients, who received a conditioning regimen consisting of treosulfan and others, achieved stable hematopoietic engraftment and stable donor chimerism without GVHD. The regimen with treosulfan could be an additional option when unrelated donor HSCT is considered for a patient with MPS (Schwinger et al 2014) although the donor cell engraftment into the brain might be limited with this type of transplant. The long-term observation of HSCT with treosulfan is required. Neonatal or early HSCT can be more widely spread as the main therapy for patients with MPS if treosulfan regimen is established in each type of MPS.

It should be noted that, to date, cord blood is a clinically useful source of HSCT for Hurler syndrome. Full-donor chimerism and normal enzyme levels are frequently achieved during the follow-up period (Boelens et al 2013; Staba et al 2004). Cord blood transplantation also improves neurocognitive development in children with Hurler syndrome (Staba et al 2004). In general, HSCT with cord blood has many advantages such as 1) easy procurement, 2) no risk to donors, 3) low risk of transmitting infections, 4) immune tolerance allowing successful transplantation despite HLA disparity, and 5) immediate availability (Aldenhoven and Kurtzberg 2015; Wagner et al 2009). The latter is clearly suitable for performing an early therapy. It is still critical to assess whether HSCT with cord blood might provide significant GVHD or not with more cases.

For what concerns neonatal gene therapy, animal studies on MPSs suggest that the viral and non-viral vectors have stably overexpressed within a long period of 10 years. In ex vivo gene therapy, retroviral vectors improved CNS disease in MPS I (Zheng et al 2003) and MPS IIIB (Zheng et al 2004) mice. Direct infusion of viral vectors into the brain also improved CNS disease by in vivo gene therapy (Berges et al 2006; Bosch et al 2000; Ciron et al 2006; Desmaris et al 2004; Ghodsi et al 1998). However, the efficacy of gene therapy for bone lesions remains unsolved. Several clinical trials in gene therapies for MPSs are currently underway in the United States. Phases I and II clinical studies for the therapy of Sanfilippo A syndrome using adeno-associated viral vector serotype rh. 10 carrying the human N-sulfoglycosamine sulfohydrolase (SGSH) and sulfatase-modifying factor (SUMF1) cDNAs are still ongoing (Tardieu et al 2014).

The future ideal gene therapy practice is that the defective gene is replaced with a normal sequence at its natural location. This is advantageous compared with a virally delivered gene which includes the full coding and regulatory sequences when only a small proportion of the gene is required to be changed, like a point mutation or small deletion and insertion. The expression of the partially replaced gene can be more consistent with normal cell physiology than the full gene accommodated by the viral vector.

**Fig. 2:**
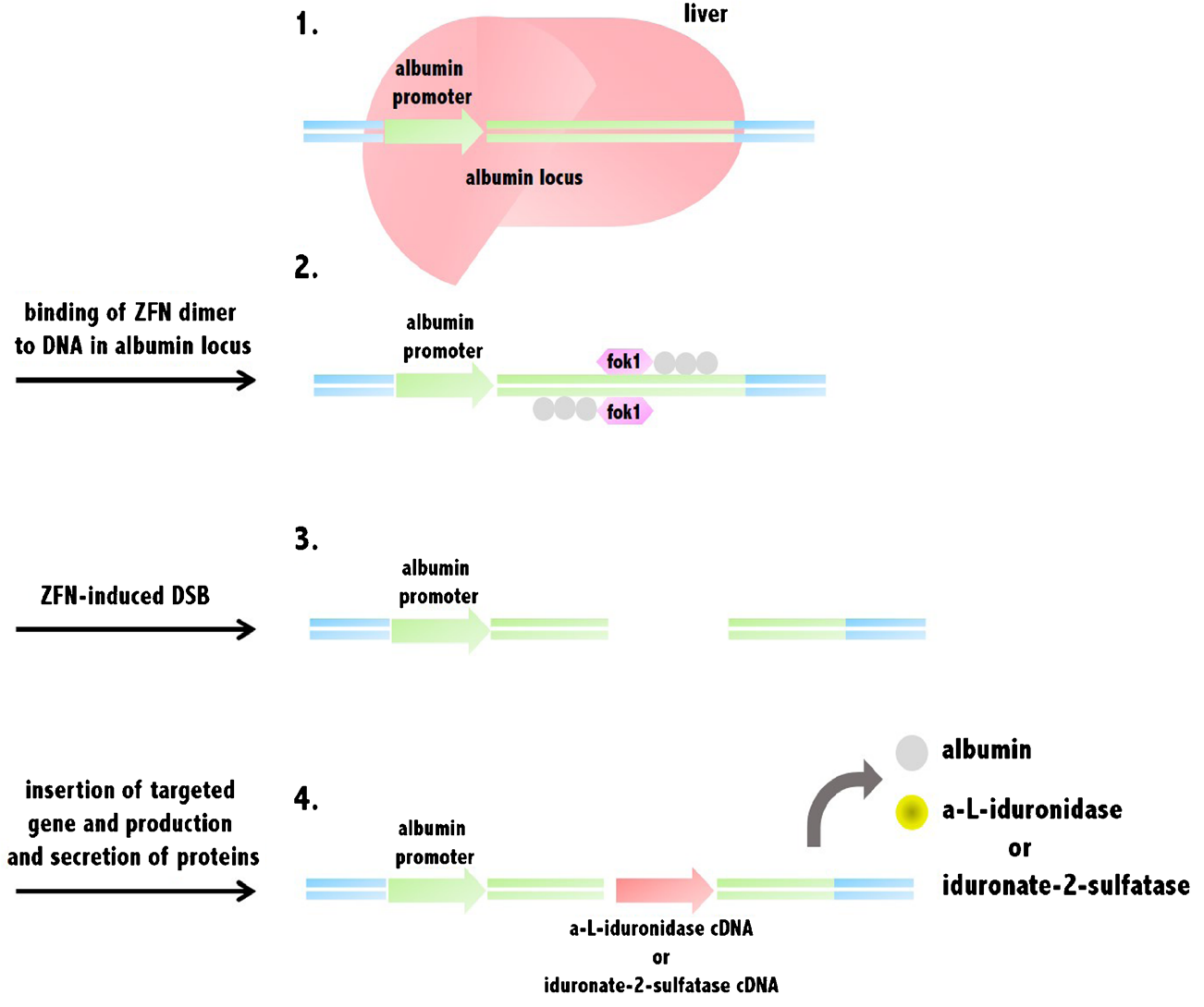
Gene editing-based therapy in human albumin locus. 1. Gene editing-based therapy for MPS I or II patients targets albumin locus sites in the liver. 2. ZFN binds to DNA sequences in the albumin locus and their fok1 nuclease domains dimerize between the binding sites. 3. ZFN induces DSB at the albumin locus in the genome of hepatocytes. 4. Alpha-L-iduronidase or iduronate-2-sulfatase genes are inserted into the albumin locus, and their proteins are continually produced from the liver into the blood. Abbreviation: ZFN, zinc finger nucleases; DSB, double-strand breaks

Gene editing, or gene editing with engineered nucleases, is a type of genetic engineering representing an innovative technology. Through this system, a DNA fragment of interest is inserted, replaced, or removed from a genome by using artificially engineered nucleases or “molecular scissors”. The nucleases generate specific double-strand breaks (DSB) at preferred sites in the genome and employ the endogenous mechanisms of homologous recombination and nonhomologous end-joining to restore the induced break point. At present, four families of engineered nucleases are in use experimentally or in clinical trials: zinc finger nucleases (ZFNs), transcription activator-like effector nucleases (TALENs), the CRISPR/Cas system (CRISPR: clustered regularly interspaced short palindromic repeats; Cas: CRISPR-associated genes), and engineered meganuclease and re-engineered homing endonucleases (Esvelt and Wang 2013; Puchta and Fauser 2014; Tan et al 2012) (Fig. 2).

For example, ZFN-induced targeting attacks defective genes at their endogenous chromosomal locations. Treatment of X-linked severe combined immunodeficiency has been administered by ex vivo gene correction with DNA carrying the IL-2 receptor common γ chain with the correct sequence (Lombardo et al 2007). However, one of the concerns about this new technology is that ZFNs may induce off-target mutations, apart from viral transductions. Many measures are under development to improve off-target detection and ensure safety prior to clinical use.

A particular use of gene editing is the in vivo protein replacement platform (IVPRP), which provides a broadly applicable genetic approach to enzyme replacement for LSDs (DeKelver et al 2015). The IVPRP applies ZFN-mediated gene editing to insert precisely normal genes into the albumin locus of liver cells in patients (Fig. 2). Successively, the normal enzyme is produced by the robust device that naturally drives albumin expression, leading to the production and secretion of the defective enzyme by the liver. At this moment, Hurler syndrome and Hunter syndrome are considered as candidate diseases, and the aim is to use this IVPRP approach to facilitate the liver to produce in patients therapeutic quantities of the normal enzymes, α-L-iduronidase and iduronate-2-sulfatase, respectively.

Non-viral vector systems such as *sleeping beauty* transposon or phiC31 recombinase-derived vector represent other approaches for safe gene transfer in MPSs (Aronovich et al 2009; Stilhano et al 2015). However, the IDUA gene expression obtained with these methods decreases over time due to the immune response and the metilation of the CAG promoter.

Further long-term clinical studies are needed to evaluate the therapeutic efficacy of gene therapy for MPS patients.

**Fig. 3:**
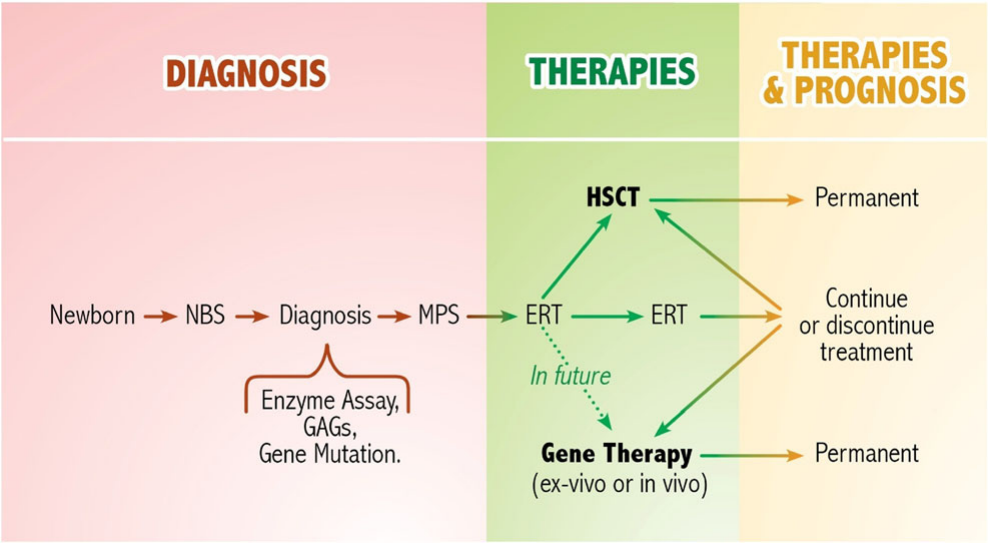
Potential scheme of future neonatal therapy for mucopolysaccharidoses. Currently, ERT or HSCT are established treatments and have a beneficial effect on patients with MPSs. In future, neonatal cellular and gene therapy approaches combined with ERT could be applied to affected children diagnosed at birth through the newborn screening. Abbreviation: NBS, newborn screening; GAGs, glycosaminoglycans; ERT, enzyme replacement therapy; HSCT, hematopoietic stem cell transplantation

It is also important to take into account that the clinical efficacy of any innovative cellular and gene therapy procedure is strictly dependent on providing the missing protein at a very early stage, before symptoms become apparent. In future, ERT should be envisaged in combined therapy with neonatal HSCT or gene therapy for MPS patients soon after the neonatal diagnosis (Fig. 3). In fact, the combination of neonatal ERT and delayed (5 weeks of age) BMT has already been performed in a murine model of MPS VII with promising results (Sands et al 1997).

To conclude, the perspective in the cure of MPSs should be more focused not only on the use of combined treatments, but also on the timing at which the therapies are given to patients, considering that the clinical outcome could be more favorable if the treatments are used to prevent instead of correcting the disease manifestations.
